# Effects of electrical stimulation on rat limb regeneration, a new look at an old model

**DOI:** 10.1038/srep18353

**Published:** 2015-12-17

**Authors:** Liudmila P. Leppik, Dara Froemel, Andrei Slavici, Zachri N. Ovadia, Lukasz Hudak, Dirk Henrich, Ingo Marzi, John H. Barker

**Affiliations:** 1 Frankfurt Initiative for Regenerative Medicine, Experimental Orthopedics and Trauma Surgery, J.W. Goethe University, Friedrichsheim gGmbH, Marienburgstraße 2, Frankfurt/Main, 60528, Germany; 2 Department of Trauma, Hand and Reconstructive Surgery, J.W. Goethe University, Theodor-Stern-Kai 7, Frankfurt am Main, 60590, Germany; 3 Department of Orthopedics, J.W. Goethe University, Friedrichsheim gGmbH, Marienburgstraße 2, Frankfurt/Main, 60528, Germany

## Abstract

Limb loss is a devastating disability and while current treatments provide aesthetic and functional restoration, they are associated with complications and risks. The optimal solution would be to harness the body’s regenerative capabilities to regrow new limbs. Several methods have been tried to regrow limbs in mammals, but none have succeeded. One such attempt, in the early 1970s, used electrical stimulation and demonstrated partial limb regeneration. Several researchers reproduced these findings, applying low voltage DC electrical stimulation to the stumps of amputated rat forelimbs reporting “blastema, and new bone, bone marrow, cartilage, nerve, skin, muscle and epiphyseal plate formation”. In spite of these encouraging results this research was discontinued. Recently there has been renewed interest in studying electrical stimulation, primarily at a cellular and subcellular level, and studies have demonstrated changes in stem cell behavior with increased proliferation, differentiation, matrix formation and migration, all important in tissue regeneration. We applied electrical stimulation, *in vivo*, to the stumps of amputated rat limbs and observed significant new bone, cartilage and vessel formation and prevention of neuroma formation. These findings demonstrate that electricity stimulates tissue regeneration and form the basis for further research leading to possible new treatments for regenerating limbs.

Limb loss due to disease, trauma and congenital deformities, is a devastating disability. In the United States alone there are nearly 1,7 million people living with limb loss and there are approximately 185,000 new amputations each year^1^. Current treatments include reattaching the amputated limb(s), transferring autologous tissues in the form of vascularized or nonvasculatized flaps, prosthetic devices and transplanted limb(s) from brain-dead donors^2^,^3^,^4^,^5^. While these treatments provide varying degrees of aesthetic and functional restoration, each has its own associated postoperative complications and risks (reviewed in^2^).

These drawbacks continue to motivate clinicians and scientists to search for better treatment options. The optimal solution would be to harness the body’s existing regenerative capabilities to regrow new limbs. Regeneration, i.e. the ability to restore diseased or injured body parts to their original healthy state, has fascinated scientists for ages (reviewed in^6^). It is known that less complex multicellular organisms such as sponges, cnidarians and flatworms are capable of regenerating their entire organism and that this ability is lost in higher vertebrates (reviewed in^7^). Some vertebrates, such as salamanders, frogs and zebra fish can regrow partial or complete tissues and organs^8^. However, mammals’ ability to regenerate is limited to a few exceptions like deer antlers, terminal phalanges in marsupials and rodents, and distal fingertips in young children^9^.

This generally accepted fact, that mammals do not regenerate limbs, was challenged in a series of experiments conducted in the middle of the 1900s. Several attempts were made to regrow limbs using a variety of different biochemical and biophysical stimuli, such as hypertonic salt solutions, tissue lysates, tissue/nerve UV-irradiation, and carcinogens (reviewed in^10^). However, these early attempts failed to produced limb regeneration in mammal models. More recently, investigators tried applying growth factors BMP2 and BMP7 and reported stimulating new endochondral ossification^11^,^12^.

Another method, tried in the late 1960s and early 70s, was low voltage electricity. In 1972, an Orthopedic Surgeon, Robert Becker published a landmark article in the journal Nature^13^ in which he reported that he had induced partial limb regeneration, in a rat limb amputation model, using low voltage direct current (DC) electrical stimulation. Becker based this experiment on an earlier study by SD Smith who used electrical stimulation to induce limb regeneration in a normally non-regenerating frog species, Rana pipiens^14^. Becker applied low voltage DC to the stumps of amputated rat forelimbs and reported that after 7 and 28 days he observed “blastema formation, new bone, bone marrow, cartilage, nerve, skin, muscle, and epiphyseal plate formation”. Based on these findings Becker concluded “regenerative growth can be restored in mammals by application of the appropriate levels of electrical stimulations”^15^. Libbin *et al.* later reproduced Becker’s experiments, but were more careful describing their observations, and emphasized the important role “mechanical factors” might play in the observed regenerative response^16^.

Stemming partially from this early work, electrical stimulation was subsequently developed and is used widely today in clinical applications to heal dermal wounds, promote regeneration of nerves in the peripheral and central nervous systems, and to treat a variety of different bone related diseases like osteoporosis, osteoarthrosis, nonunion fractures, and to promote the integration of implanted biomaterials in orthopedics (reviewed in^17^). In recent years a great deal of research has focused on unraveling the underlying mechanisms of electrical stimulation (ES) at a cellular and subcellular level using *in vitro* model systems. These have shown that ES influences stem and progenitor cell behavior, increasing cell proliferation, differentiation, matrix formation and migration. All of these cell functions are known to play key roles in tissue regeneration (reviewed in^18^).

In contrast to the above cited *in vitro* studies, in the present study we delivered low voltage direct current (DC) electrical stimulation to the stumps of amputated rat forelimbs and used histology and immunohistochemistry to assess the resulting healing and regeneration response.

## Results

Electrical stimulation devices were well tolerated by all animals in all groups with no detectable side effects (weight gain or loss, signs of low vitality, infection or tumor formation) at the 7 and 28 day assessments. Three animals died immediately following surgery due to problems with anesthesia.

### New tissue formation

**Figure 1:**
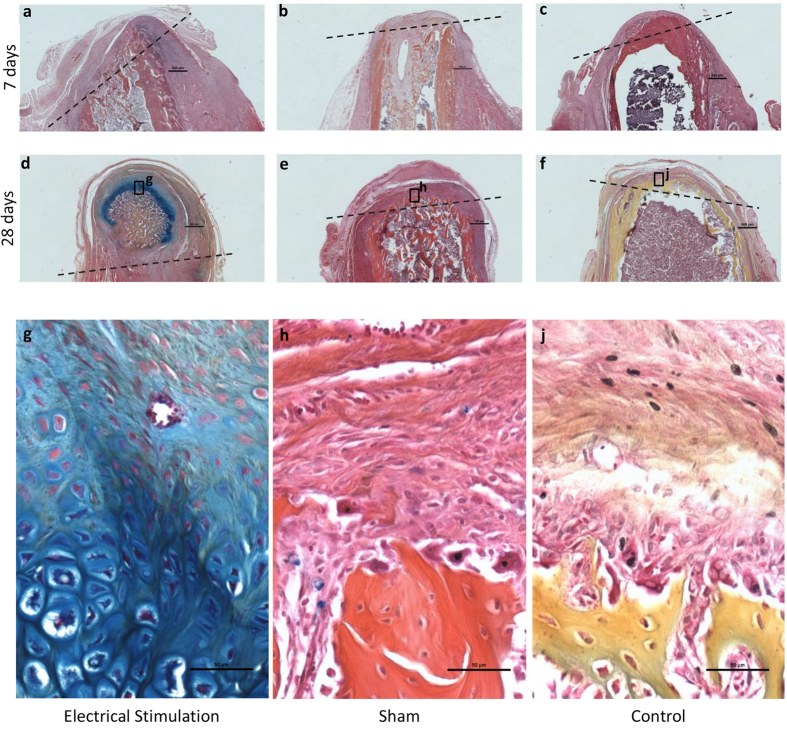
Longitudinal histological sections of rat limb stumps. All sections were stained with AB&OG. Approximate level of the amputation site is indicated with black dotted lines (**a–f**). Top row are electrically stimulated (**a**), sham (**b**) and control (**c**) stumps at 7 days after amputation (scale bar = 500 μM). Middle row are electrically stimulated (**d**), sham (**e**) and control (**f**) stumps at 28 days after amputation. Bottom row are high magnification (20x) (scale bar = 50 μM) of electrically stimulated (**g**), sham (**h**) and control (**j**) selected sites (black box) on stumps at 28 days after amputation. Significant cartilage formation is seen in the electrically stimulated (**g**) histological section versus scar formation in the sham (**h**) and control (**j**) sections.

The histological response in stump tissue distal to the amputation is shown in Fig. 1(a–j). No visible signs of inflammation were noted in any of the limb stumps at the time of harvest at 7 or 28 days, independent of the treatment received. In ES treated limb stumps the bone marrow cavity remained open on day 7, and closed with a significant accumulation of new cartilage and bone tissue at 28 days. In contrast, in non-stimulated control and sham stumps a thin layer of new tissue formation was observed covering the bone marrow cavity at the amputation site at 7 and 28 days.

**Figure 2:**
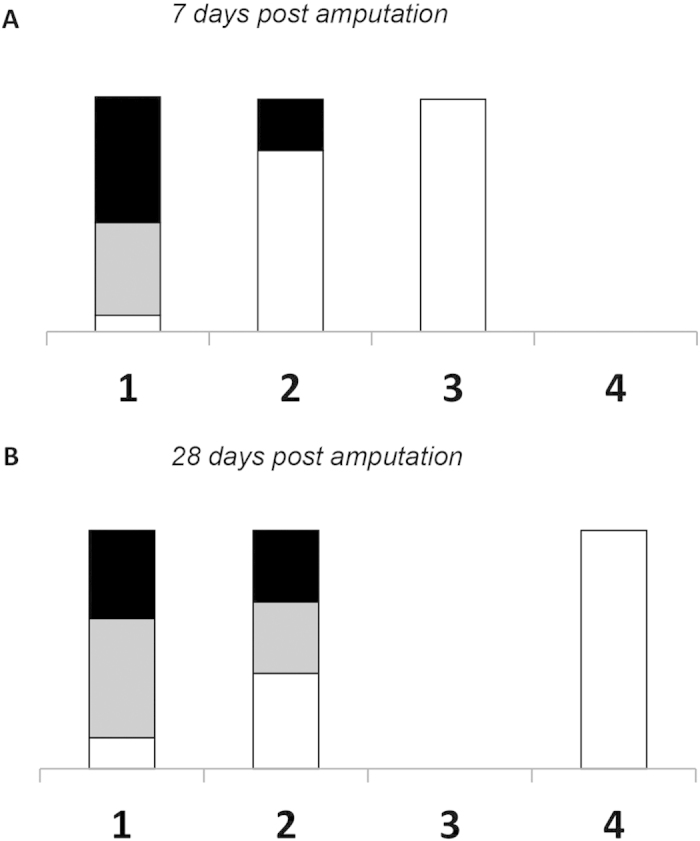
Scores indicating osteocartilaginous formation in rat limb stumps. Osteocartilaginous formation in electrically stimulated (white), sham (gray) and control (black) stumps at 7 (**a**) and 28 (**b**) days after amputation. Scores derived from a scale of +1 to +4, where +1 = no growth; +2 = growth <100 μM; +3 = growth between 100 and 1,000 μM, and +4 = growth >1,000 μM.

**Figure 3:**
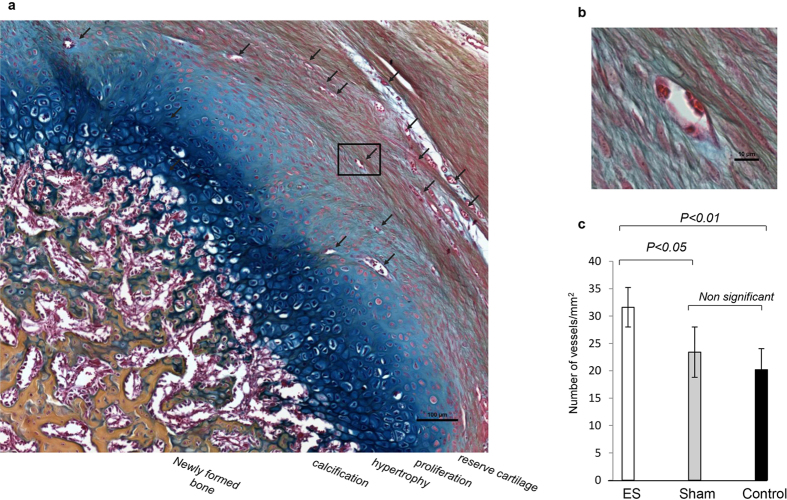
High magnification longitudinal histology section of electrically stimulated rat limb stump 28 days post amputation. (**a**) Osteochondral ossification and vascularization (AB&OG staining, 20x, scale bar = 100 μM). Vascular ingrowth (black arrows). Reserve, proliferating cartilage, hypertrophy, calcification and newly formed bone areas are visible. (**b**) High magnification (40×; scale bar = 10 μM) of new vessels. (**c**) Graph indicating number of new vessels in 3 × 1 mm^2^ square areas in rat limb stumps in all 3 groups 28 days post amputation. Significantly more new vessels are visible in electrically stimulated compared to sham (p < 0,05) and control (p < 0.01) stumps.

Various stages of new tissue (bone and cartilage) formation were observed in ES treated limb stumps. In 2 animals 100–1,000 μM of cartilage deposition (score of 3) was observed at the amputation on day 7, and in 4 animals more than 1,000 μM of cartilage deposition and woven bone formation (score of 4) was seen at 28 days (Figs 1d,g and 2a,b). In these latter 4 animals an area of organized osteocartilaginous growth was observed at the distal tip of the stump corresponding to lengths of new tissue growth measuring 3.5, 2.5, 1.5 and 1.5 mm (Fig. 1d). Reserve, proliferating, and hypertrophied cartilage, calcification and newly formed bone were visible in this area (Fig. 3a).

The stumps of all the control and sham treated animals had no (score of 1), or minimal (score of 2) cartilage deposition, and the bone marrow cavity at the amputation was closed at 7 and 28 days. In the stump of 1 sham treated rat a small amount of bone formation was detected (score of 2) in close proximity to the silver wire electrode. The appearance of this bone differed from that seen in the ES treated stumps, in its location and in the absence of osteocartilaginous formation (Fig. 1b). New muscle formation was not observed in any of the stumps, regardless of the treatment received.

### New vessel formation

Areas of new vessel formation in the stump tissue distal to the amputation were observed in limb stumps at 28 days (Fig. 3a,b). The presence of new vessel growth was significantly higher in the ES treated stumps compared to that seen in the control (P < 0.05) and sham (P < 0.01) treated stump tissue. The difference between control and sham treated stumps was not significant (Fig. 3c).

### New nerve formation

**Figure 4:**
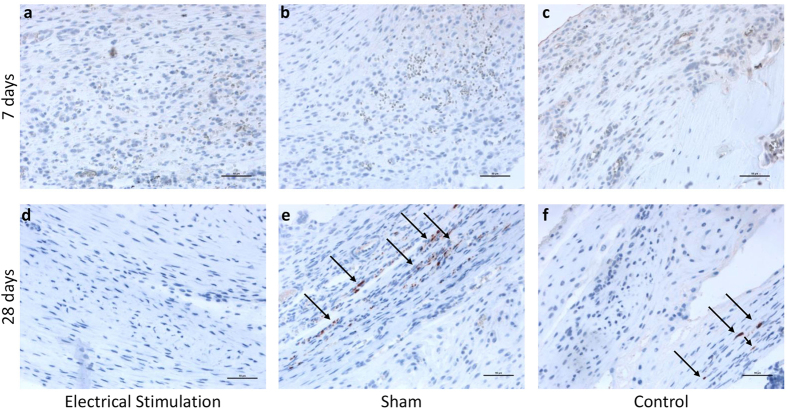
Neuroma formation in rat limb stumps at 7 and 28 days post amputation. (Neurofilament protein staining, 20× magnification, scale bar = 50 μM). (**a–c**) No signs of neuroma formation in electrically stimulated (**a**), sham (**b**) and control (**c**) stumps 7 days post amputation. Neuroma formation (arrows) in sham (**e**) and control (**f**) stumps and no neuroma formation in electrically stimulated stump tissue (**d**) 28 days post amputation.

No new nerve formation was detected in any of the three groups. However, at 28 days in the control and sham treated animals we observed histological findings consistent with neuroma formation in tissue at the distal end of the stump. These formations appeared as haphazardly arranged nerve fascicles (Fig. 4e,f). In contrast, no such neuroma formation was detected in ES treated stump tissue (Fig. 4a,d).

### Cell proliferation

Cell proliferation, as determined by the presence of BrdU positive cells, was higher in ES treated stumps than in control and sham treated tissues at day 7. At 28 days no difference was detected between the 3 groups (data not shown).

## Discussion

Intra- and extracellular electrical fields play an essential role in regulating cellular behavior, both in embryonic development and in healing and regeneration. Several examples of this have been documented in the literature. It has been shown that disrupting normal electrical fields in tissues surrounding the neural tube during chick embryogenesis causes severe developmental deformities; a cut in the skin short-circuits transepithelial potential differences and gives rise to injury current flow that plays an important role in initiating dermal healing; immediately following amputation of a newt limb elevated levels of electrical current emanate from the amputated stump for 10–14 days, and as the limb regrows levels decrease to pre-amputation levels (reviewed in^19^). These observations underscore the key role electrical signals play in regulating tissue development, healing and regeneration^20^.

In the middle of the last century, these observations led researchers to experiment with the application of electrical current to stimulate healing and regeneration in bone, skin, nerves and even whole limbs (reviewed in^19^,^21^). These studies led to the development of several clinical treatments that deliver external electrical stimulation to enhance healing and regeneration in bone, soft tissue, and spinal cord (reviewed in^22^,^23^,^24^). However, in spite of these positive effects observed in individual tissues, regeneration of whole limbs or digits has not yet been induced in mammals. In the present study we investigated the effects of DC electrical current on whole limb regeneration in a rat limb amputation model.

We selected this model because it is a mammal that is known to have minimal regenerative capacity after injury, thus assuring that any regenerative response observed would be due to the electrical current delivered. In addition, we chose the rat limb because it is made up of composite tissues so that we could study the effect electrical stimulation has on healing and regeneration in multiple tissues together. Finally, this model has been used and validated by other investigators in previous experiments^16^,^25^,^26^.

Using the amputated rat limb model we demonstrated that electrical stimulation caused cell proliferation at 7 days, followed by significant osteocartilaginous growth and cartilage formation at 28 days. This finding, also reported by Becker and Libbin^15^,^16^, does not occur in normal healing in postnatal mammals^27^, and instead has the appearance of organised tissue growth seen in a regenerative response. In contrast to this observation, tissues in our control and sham stumps had the appearance of normal healing tissue, with low levels of osteogenesis and bridging with bone at the cut surface. This observation was also reported by Becker^27^.

Contrary to Becker, however, we did not observe “blastema formation, regrowth of complete humerus, new organized skeletal muscle formation or nerve regeneration”^25^. Libbin *et al.*, who reproduced Becker’s experiments, like us, did not observe blastema formation or nerve ingrowth, however, like Becker, did observe myogenesis^16^. Reasons for these differences could be due to differences in the animals or the surgical amputation procedure, however, more likely are explained by the more advanced analysis methods and equipment we used to prepare and analyze the histological samples.

Our results showed that DC electrical stimulation significantly increases vascularization. While not reported by Becker or Libbin, in more recent studies this finding was reported by Ud-Din, S *et al.* who showed that pulsed electrical stimulation increases vascularization and therefore healing in injured human skin^28^. In addition Bai *et al.*, in *in-vitro* experiments found that DC electrical stimulation induced a significant angiogenic response in vascular endothelial cells, upregulating angiogenic factors through activation of VEGF receptors^29^. The key role that vascularization plays in many important physiological and pathological processes, including bone development and repair, is well documented^30^. In fact the absence of sufficient blood supply is a major cause of impaired bone healing^31^ and non-union^32^. This effect alone could be one of the major contributors to the improved healing seen with the different clinical treatments using electrical stimulation (reviewed in^22^,^23^,^24^).

In the present study, in ES treated limb stumps we observed that the bone marrow cavity remained open on day 7, while in sham and control animals the cavity was closed with a thin layer of new tissue at day 7. At 28 days the bone marrow cavity of ES stumps was closed with new cartilage and bone tissue while the control and sham stumps had the same thin layer of tissue seen at day 7. This delay in closure of the bone marrow cavity in ES treated stumps might suggest that ES contributes to a shift in the balance towards continued proliferation and regeneration versus the scar-like formation seen in the control and sham treated stumps. This is an important observation that we will pursue in future studies using this model.

Another important finding we observed was the inhibition or prevention of neuroma formation in our ES treated limb stumps. Neuromas are a tumor-like thickening of severed nerve stumps in the region of a scar after amputation and are thought to be caused by the disorganized growth of axon cylinders into proliferating granulation tissue. Neuroma formation inhibits nerve regeneration after injury and in amputee patients can cause severe pain, tingling sensation and significant loss of function^33^. In the limb stump tissue of our sham and control animals we saw important neuroma formation while in electrically stimulated stumps no signs of neuroma formation were detectable. While our histological analysis was insufficient to determine the mechanism of this effect a possible explanation could be paracrine modulation of Wallerian degeneration and overall regenerative response by MSC. This was recently described by Gärtner, A *et al.* in which case they studied end-to-end sciatic nerve repair in a rat model^34^. Because of the important potential clinical implications of this finding for treating amputee patients we are actively pursuing this effect of ES in ongoing studies in our laboratory.

Based on our results we hypothesize that in our rat limb amputation model electrical stimulation causes the observed effect by stimulating bone marrow stem/progenitor cells to generate highly vascularized osseocartilaginous centers in the zone of injury. This hypothesis is supported by our own *in vivo* and *in vitro* observations and those of others. *In vivo* we observed that the tissues that demonstrated the greatest amount of proliferation (bone, cartilage, vessels) were of MSC and EPC origin. *In vitro* we have shown that ES increases osteogenic differentiation of MSC (data submitted for publication elsewhere). Finally others have shown *in vitro* and *in vivo* that ES stimulates MSC and EPC proliferation, differentiation and migration^35^^–^37. In *in-vivo* experiments ES has been shown to increase the number of osteoblasts^38^,^39^, increase EPC migration, elevate VEGF levels, and activate VEGF receptors^29^. Finally, increasing evidence suggests that MSC may play an important role in tissue regeneration through the secretion of soluble trophic factors that enhance and assist in repair by paracrine activation of surrounding cells. Preclinical and clinical findings have demonstrated that MSC have the ability to migrate to specific sites of injury or regeneration where they modulate the immune and inflammatory responses and mobilize intrinsic cell reservoirs through a series of distinct paracrine mechanisms^40^. While it was beyond the scope of the present study to determine the effect of ES on MSC *in vivo*, in future studies, we plan to tag MSC in ES treated rat limbs and measure their presence and activity.

In conclusion, this study demonstrated that low voltage DC electrical stimulation promotes healing and regeneration of specific tissues in the stump of our rat limb amputation model. This effect was most pronounced in osteocartilaginous and vascular tissue. In addition, we observed the inhibition of neuroma formation. In future studies, in order to better define the underlaying mechanisms causing the observed effects we plan to identify specific gene expression and pathways affected by electrical stimulation in this model. These studies will allow us to better understand the role of ES in mammal limb regeneration and in doing so help to improve and expand its use in the clinical setting.

## Methods

All animal experiments were performed in accordance with guidelines established by our animal care and oversight committee at the Johann Wolfgang Goethe-University in Frankfurt/Main, Germany and were approved by the Veteranary Department of the Regional Council in Darmstadt, Germany (Regierungspräsidium Darmstadt, Veterinärdezernat, Wilhelminenstraße 1–3) (Project No. F3/25).

In order to assess the effects of electrical stimulation on limb tissue regeneration the right forelimbs of 48 Sprague Dawley rats (Charles River Labs Int., Germany) (age = 5 weeks; weight = 100–150 g) were amputated and the limb stumps were treated with: 1) Electrical stimulation + active device (n = 16), 2) No electrical stimulation + inactive device (n = 16), and 3) No electrical stimulation + no device (n = 16). Table 1 shows the treatment, the number and the distribution of animals.

### Electrical stimulation device

**Figure 5:**
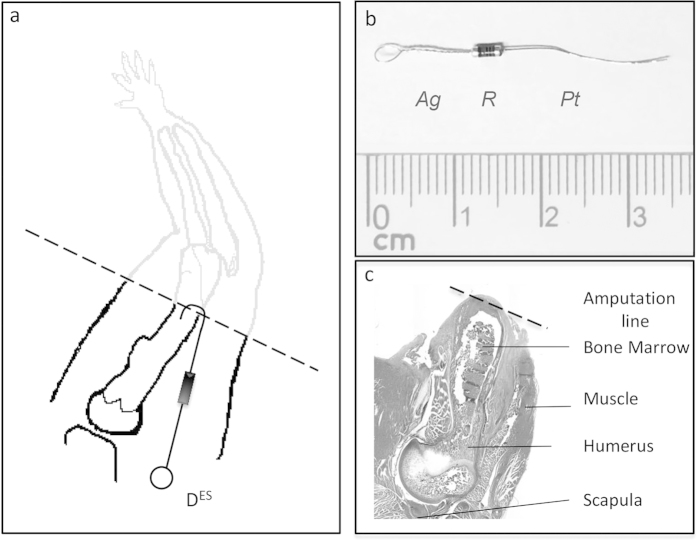
Drawing and photos of histological section of amputated rat limb stump, and ES device. (**a**) Drawing of rat limb amputation with the amputation site (black dotted line) and implanted electrical stimulation device (D^ES^). (**b**) Photo of the electrical stimulation device with a silver wire electrode loop (Ag) and platinum wire electrode (Pt) interposed with a 10 MΩ resistor (R). (**c**) Photo of longitudinal histological section of amputated limb stump, with the amputation site (black dotted line) and labeled bone marrow, muscle, humerus and scapula bones.

Electrical stimulation (ES) was applied using a purpose-built bimetallic device consisting of platinum and silver wire electrodes with their proximal ends laser-welded to a 10 MΩ resistor (Fig. 5b). The platinum electrode measured 1,3 cm in length with a diameter of 0,15 mm, and the silver electrode was 3 cm long with a diameter of 0,15 mm and had a loop tied at the free end (Junker-Edelmetalle, Waldbüttelbrunn, Germany). The welds were reinforced with quick drying, 2-component epoxy resin glue (UHU, Germany) and the electrode-resistor union was completely encapsulated/isolated in medical grade silicone (RTV-coating, Dow Corning, USA), leaving only the silver loop and 4 mm of the distal end of the platinum electrode exposed. Prior to surgical implantation devices were sterilized in 95% Ethanol for 1 hr and exposed to UV light for an additional 1 hr and washed with sterile PBS solution (Sigma-Aldrich, Germany). In the sham group a 2,5 cm long piece of looped silver wire served as the “inactive” device.

### Limb amputation and ES device implantation

Prior to surgery animals received prophylactic antibiotics (0.2 ml procaine penicillin containing 60,000U). While under intraperitoneal general anesthesia (Ketamine/Xylazine 100 mg/10 mg/kgKG) their right limbs were shaved and cleaned with antiseptic fluid and under aseptic conditions the brachial artery was identified, dissected free and ligated through a skin incision at the medial aspect of the right upper forelimb. A circumferential skin incision was made on the forelimb exposing the humerus bone which was cut using a guillotine technique with surgical bone-cutting shears 1 cm proximal to the elbow joint, to assure it did not interfere with the distal growth plate.

Electrical stimulation devices (Fig. 5a,b) were implanted immediately after amputation. In animals whose limb stumps received electrical stimulation (Group 1) the ES device and the silver electrode were placed in the musculature of the right shoulder of the amputated limb and secured with sutures to the deltoid fascia through the silver electrode loop. The platinum electrode was bent and inserted approximately 2 mm into the medullary cavity of the humerus bone at the amputation site. Sham animals (Group 2) received a single 2,5 cm long looped silver wire, which was inserted 2 mm into the medullary cavity and sutured proximally. The limbs of the control animals (Group 3) were amputated, but neither device nor electrodes were implanted. In all 3 groups the skin was closed over the limb stump with a continuous intradermic suture (4-0 Prolene, Ethicon, Germany) and animals received postoperative enrofloxacin antibiotics (Baytril, Bayer, Germany) via intraperitoneal injection. After surgery animals were monitored until they recovered from anesthesia and daily for complications or signs of pain and discomfort. Animals were housed in separate cages in a light (12 hr light – 12 hr dark), temperature (20–24° C) and airflow controlled room and were given free access to food and water. Animals were euthanized (CO_2_ inhalation) at 7 and 28 days post amputation and weighed. The limb stumps were collected and examined macro- and microscopically for signs of infection or tumors. The limb stump specimens were fixed in Zinc-Formal-Fixx, Zinc-Formal-Fix x, (Thermo scientific, USA) for 24 hrs and stored for subsequent histomorphometric and immunohistological analysis.

### Histomorphometry

Fixed stumps were decalcified for 14 days in a solution containing 10% EDTA/Tris-H Cl pH 7.4 (Sigma) and were paraffin embedded. Sections (7 μm thick) were taken parallel to the long axis of the humerus and stained with Alcian Blue - Orange G-Hematoxilin-Eosin (AB&OG)^41^. All histological sections were analyzed for new tissue formation, new vessel and nerve formation, and cell proliferation. Analysis and quantitative evaluations were done using light microscopy (Large image scanning, Ti-E, Nikon GmbH, Germany) and image analysis software (NIS-Elements 4.4, Nikon GmbH, Germany).

### Assessment of new tissue formation

In order to quantify the amount of new tissue formation a scoring method was used on standardized images of histological sections using an arbitrary scale of + 1 to + 4, where;

+1 No growth, closure of the bone marrow cavity at the amputated bone end,

+2 <100 μM cartilage deposition at the amputated end,

+3 100–1,000 μM cartilage deposition at the amputated end, and

+4 >1,000 μM cartilage deposition and woven bone formation at the amputated bone end

The distribution of electrically stimulated, sham and control samples for each score value was analyzed graphically by % stacked column graph (X-cell, Microsoft office for Windows).

### Assessment of vascularization

The number of vessels, in a standardized field of a histological section, stained with AB&OG, was counted using light microscopy and image analysis software (NIS-Elements 4.4, Nikon GmbH, Germany). Assessments were performed in blinded specimens examined in random order. Three 1 mm^2^ areas at the distal end of the stump where identified and vessels were counted by an independent observer (main measure/count option), blinded to the group setup. The mean number of vessels within the 3 areas was calculated and the means were subsequently used for statistical analysis.

### Assessment of neuroma formation (Immunohistochemistry)

Paraffin embedded, 7 μm thick sections of decalcified specimens were incubated with monoclonal mouse anti-human Neurofilament protein antibodies, which cross react with both human and rat neurofilament proteins (Clone 2F11, culture supernatant, 1:100; DAKO, Germany). An Isotype identical (IgG1) non-specific mouse antibody served as a negative control (eBioscience, Germany). For signal detection, an EnVision+ System-HRP (AEC) kit (Dako, Germany) was applied. Finally, a counterstain with hematoxylin was performed. Three slides per animal were analyzed using light microscopy (at 10x) and image analysis software.

### Assessment of cell proliferation (*in vivo* BrdU incorporation assay)

Cell proliferation was measured with the thymidine analog BrdU (5-Bromo-2′-deoxyuridine) following its incorporation into newly synthesized DNA and its subsequent detection with an anti-BrdU antibody. Animals were administered sterile BrdU labeling reagent (intraperitoneal 1 ml/100 g body weight) (Life Technologies). Injections were performed on day 27 (1 day prior to harvesting the 28 day stumps) in 8 experimental animals, 8 controls, and 8 sham animals (Table 1) and at day 6 (1 day prior to harvesting the 7 day stumps) in 2 control and 2 experimental animals (Table 1). Stumps were collected, fixed, and decalcified as described above. Paraffin embedded 7 μm thick sections of the decalcified specimens were incubated with biotinylated monoclonal anti-BrdU antibodies according to the manufacture’s protocol (BrdU staining kit, Invitrogen). Quantitative evaluation of BrdU labeled cells was performed in a standardized image of histological sections using light microscopy (Ti-E, Nikon GmbH, Germany) and image analysis software (NIS-Elements 4.4, Nikon GmbH, Germany). Assessments were performed with blinded specimens examined in random order. Three 0,5 mm^2^ areas were chosen in the distal end of the stump. Labeled cells were marked by an independent observer (main measure/count option), blinded to the group setup, and the mean number of positive stained cells within the 3 areas was calculated. These means were subsequently used for statistical analysis.

### Statistical analysis

Data were analyzed using Unifactorial analysis of variance ANOVA, (group number = 3), BiAS for Windows^TM^ version 11.0 software (http://www.bias-online.de). Cohen’s effect size d: d = 0,2 minimal effect; d = 0,5 middle effect; d > 0,8 bigger effect. Data are presented as mean ± SD and significance level was set at P < 0,05.

## Additional Information

**How to cite this article**: Leppik, L. P. *et al.* Effects of electrical stimulation on rat limb regeneration, a new look at an old model. *Sci. Rep.* **5**, 18353; doi: 10.1038/srep18353 (2015).

## Figures and Tables

**Table 1:** Experimental design and distribution of animals per group.

	Days post- amputation	Number of animals per group		
		Electrical stimulation	Sham	Control
**Number of rats in 28 & 7 day groups**	28	8	8	8
	7	8	8	8
**Measurements Performed**				
Histomorphology*	28	7	8	7
	7	8	7	8
Vascularization*	28	5	5	5
	7	5	5	5
Neuroma formation*	28	5	5	5
	7	5	5	5
Cell proliferation*	28	5	5	5
	7	2	–	2

^*^Minimum of 5 histological samples were analyzed for each animal.
